# Impacts of mycorrhizal types, tree diversity, and species identity on the soil microbial genomic functional potential in temperate forests

**DOI:** 10.1128/spectrum.00295-25

**Published:** 2025-11-03

**Authors:** Hafeez Ul Haq, Bala Singavarapu, Olga Ferlian, Henriette Christel, Simone Cesarz, Nico Eisenhauer, Helge Bruelheide, Tesfaye Wubet

**Affiliations:** 1 Department of Community Ecology, Helmholtz Centre for Environmental Research UFZ 28342, Halle, Germany; 2 German Centre for Integrative Biodiversity Research (iDiv) Halle-Jena-Leipzig 530625 , Leipzig, Germany; 3 Institute of Biology/Geobotany and Botanical Garden, Martin Luther University Halle Wittenberg 9176, Halle, Germany; 4 Aquatic Geomicrobiology, Institute of Biodiversity, Friedrich Schiller University Jena 9378, Jena, Germany; 5 Institute of Biology, Leipzig University 9180, Leipzig, Germany; University of Mississippi, University, Mississippi, USA

**Keywords:** microbial diversity, community composition, mycorrhizal type, tree diversity, C, N, P cycling genes, genomic functional potential, C:N:P stoichiometry

## Abstract

Soil microbial genomic functional potential refers to the community’s collective genetic capacity to mediate nutrient cycling processes involving carbon (C), nitrogen (N), and phosphorus (P). While the drivers of microbial community composition have been extensively studied, particularly in forest ecosystems, the effects of tree species identity, mycorrhizal type, and their mixture along tree diversity gradients on microbial genomic potential remain poorly understood. We predicted microbial genomic functional potential for carbon (C), nitrogen (N), and phosphorus (P) cycling genes, along with their ratios (C:N, C:P, N:P), using fungal ITS2 and bacterial 16S rRNA metabarcoding data from the rooting zone soil samples of eight tree species growing in the MyDiv experiment. We assessed the effects of a tree species identity, plot mycorrhizal type (mono: AM or ECM and mixed: AE = AM + EcM), and tree diversity levels (one, two, four) on the microbial C, N, and P cycling genomic potential and their ratios. We also tested the relationship of microbial diversity, microbial beta-diversity, and soil C:N:P stoichiometry with microbial C, N, and P cycling genomic potential and their ratios. Two-way ANOVAs showed that mycorrhizal type mixture consistently influenced the fungal and bacterial C, N, and P cycling genomic potential and their ratios, while tree diversity significantly affected fungal C and P genomic potential, as well as bacterial C and C:P ratio genomic potentials. Tree species identity impacted fungal genomic potential, particularly in AM and AE types, whereas bacterial functional potentials were significantly linked to tree diversity across AM, AE, and EcM plots. Fungal richness decreased significantly with C and P genomic potential in EcM plots but increased with N genomic potential in AM plots, showing contrasting effects on genomic potential ratios, while bacterial richness showed no significant relationship. Fungal and bacterial beta-diversity were both positively associated with C and P cycling genomic potentials in EcM and AE plots. The C:N and C:P genomic potential ratios increased, and N:P ratios decreased with increasing fungal beta-diversity in EcM and AE plots. Similar patterns were observed for bacterial genomic potential across all AM, EcM, and AE plots. Overall, soil C:N:P stoichiometry showed weak and non-significant correlations with microbial C, N, and P cycling genomic potential. Our findings highlight that plot mycorrhizal type mixture, tree species identity, and tree diversity shape the microbial C, N, and P cycling genomic functional potential. These results also underscore that community turnover, taxonomic replacement, and compositional restructuring of taxa, rather than merely overall richness, are the primary drivers of functional diversification in carbon, nitrogen, and phosphorus cycling among microbial communities.

**IMPORTANCE:**

Our study suggests that forest management strategies fostering microbial community diversity through mixing tree species with different mycorrhiza types and increasing tree diversity could enhance soil microbial functions, increase nutrient cycling, and improve forest ecosystem resilience and productivity.

## INTRODUCTION

The availability and balance of carbon (C), nitrogen (N), and phosphorus (P) in soil play a vital role in regulating plant growth, nutrient cycling, and overall ecosystem health. Soil microorganisms are central to terrestrial ecosystems, as they drive biogeochemical cycles, support biodiversity, and maintain essential ecosystem functions (1, 2). These microbes enhance plant nutrient uptake both directly through mechanisms such as mycorrhizal associations and nutrient immobilization and indirectly via the decomposition of organic matter and nutrient cycling mediated by extracellular enzymes (3, 4). Nutrient stoichiometry in soils influences both soil properties and broader ecosystem functions, thereby shaping microbial community composition and nutrient dynamics within the plant-soil interface (5, 6). In turn, microbial activity shapes these systems by regulating nutrient availability through processes such as decomposition, nutrient immobilization, and transformation (7). For example, elevated soil C:N and C:P ratios indicate greater inputs of carbon-rich organic matter utilized by microbes, while lower N:P ratios point to a greater presence of phosphorus-rich compounds, such as ribosomes, which are essential for protein synthesis (8).

Beyond nutrient concentrations and their ratios, growing attention has been given to the functional genomic potential of soil microbial communities—their collective genetic capacity to carry out processes related to C, N, and P cycling (9, 10). A higher abundance of genes encoding enzymes, such as glycosaminidases and phosphatases, can boost a microbial community’s ability to decompose organic matter, recycle nutrients, and sustain ecosystem functions (11). The presence of genes related to nitrogen cycling, phosphorus solubilization, and carbon breakdown directly affects how much of these vital nutrients are accessible in soil (12, 13). Microbial communities with diverse and abundant functional gene profiles are better equipped to support robust nutrient cycling and ecosystem resilience (14). Thus, the genomic functional potential of soil microbes is tightly linked to how much carbon, nitrogen, and phosphorus is accessible in the soil environment. Soils with higher carbon content may harbor microbial communities with enhanced capabilities to break down carbon-rich organic matter (15), while soils with a more balanced C:N and C:P ratio might favor microbial pathways that accelerate nitrogen and phosphorus turnover (10, 16). For example, soils with high C:P or N:P ratios have been shown to constrain microbial growth due to phosphorus limitation, leading to increased expression of phosphorus-acquisition genes (*phoD*, *ppx*) and a community shift toward oligotrophic taxa (e.g., Acidobacteria, Verrucomicrobia) adapted to low-nutrient environments (12, 17). While it is well established that C:N:P stoichiometry shapes microbial community structure and influences processes, such as nutrient cycling and organic matter decomposition (7, 8, 18), how these nutrient ratios (C:N, C:P, N:P) relate to the abundance and functional balance of microbial genes involved in C, N, and P cycling is still not fully understood.

In terrestrial ecosystems, over 80% of plants engage in symbiotic relationships with fungi, primarily either with arbuscular mycorrhizal (AM) or ectomycorrhizal (EcM) fungi (19, 20). AM-associated plants tend to exhibit enhanced uptake of inorganic nutrients, whereas EcM-associated plants rely more on the mineralization of organic matter for nutrient acquisition (18, 21). These functional differences also influence the composition and activity of their associated microbial communities. AM fungi release labile carbon compounds that promote the growth of microbial taxa involved in organic matter decomposition and phosphorus solubilization, such as *Pseudomonas* spp (22, 23). Conversely, EcM fungi secrete oxidative enzymes to degrade complex organic substrates, altering soil pH and carbon dynamics, which in turn favors bacterial taxa like *Actinobacteria* that specialize in metabolizing nitrogen-rich organic residues (24, 25). These cross-kingdom interactions suggest that mycorrhizal type not only determines the fungal partner but also shapes the broader rhizosphere and hyphosphere microbial communities and their functional potential for nutrient cycling. As a result, AM and EcM fungi exhibit distinct genomic traits aligned with their ecological roles in carbon, nitrogen, and phosphorus cycling (26, 27). While AM fungi are particularly efficient at phosphorus uptake, they depend heavily on co-occurring microbial partners in the rhizosphere and hyphosphere for P mobilization and cycling (28). In contrast, EcM fungi possess a more complex genomic repertoire, including enzymes such as proteases and chitinases, enabling them, along with their microbial partners, to access organic forms of carbon and nitrogen through the breakdown of complex organic matter (28). Due to these functional distinctions, AM and EcM fungi contribute uniquely but complementarily to the soil C, N, and P dynamics, plant nutrition, and ecosystem productivity (26, 27). Despite recent advances, the interactive effects of tree mycorrhizal type, mycorrhizal mixtures, tree species identity, and tree diversity on the rhizosphere microbial communities (5, 6) remain a substantial knowledge gap regarding how these shifts in microbial community composition translate into changes in the genomic functional potentials for C, N, and P cycling.

To address these knowledge gaps, we used the MyDiv experimental platform (29), where tree species of two mycorrhizal types (AM and EcM) grow in one-, two-, and four-species mixtures in either mono (AM or EcM)- or mixed (AE, where AM and EcM trees grow together)-mycorrhizal type plots (5). We used V4 16S rRNA and ITS2 fungal ITS rDNA amplicon sequencing data generated from target tree rooting zone soil samples of eight tree species (5) (Fig. S1). We employed PICRUSt2 (30), a bioinformatics tool that predicts the functional potential of microbial communities based on 16S rRNA and ITS amplicon sequencing data to infer microbial genomic potentials linked to carbon (C), nitrogen (N), and phosphorus (P) cycling genes (31). Despite its limitations, such as reduced accuracy due to the underrepresentation of soil taxa in reference genomes and potential biases in functional inference (30), PICRUSt2 was used here as a cost-effective, scalable method for exploratory functional profiling, allowing us to link microbial community data to C, N, and P cycling gene predictions. Then, the following research questions and hypotheses were evaluated.

Do mycorrhizal type mixtures, tree species identity, and neighbor tree diversity influence the microbial genomic functional abundance of the rooting zone of a target tree species? Since AM and EcM trees shape their rhizosphere soil microbiome diversity and composition differently (28), we expected that microbial genomic functional abundance would be mainly affected by tree species identity and the co-existing tree species mycorrhizal type mixture, with the effect diminishing as tree diversity increases (2). Could soil microbial diversity and community composition serve as predictors of the microbial genomic functional abundance? Owing to the fact that microbial genomic potential is predicted from the pool of the rhizosphere microbiome, and that trees with different mycorrhizal types have differences in nutrient acquisition strategies and nutrient cycling (28, 32), we assume that the alpha and beta-diversity measures serve as predictors of the C, N, and P cycling gene abundances. We expect that these relationships will be mainly affected by mycorrhizal type mixture than species identity and tree diversity. Consequently (3), we expect that the identity of the dominant microbial taxa contributing to the C, N, and P cycle genes varies with mycorrhizal type mixture and tree diversity level (4). Soil organic matter availability and soil C:N:P stoichiometry are known to be affected by tree species identity, the mycorrhizal type of co-existing tree species, and stand tree diversity (5, 6). Thus, we assessed the relationship between soil C:N:P stoichiometry and the respective C:N:P microbial genomic functional gene abundances and ratios. We expected a strong relationship between the soil nutrient variables and the microbial genomic functional abundance.

## RESULTS

### Influence of mycorrhizal type mixture, tree diversity, and tree species identity on microbial functional genomic potential

The two-way ANOVA results revealed a consistent effect of plot mycorrhizal type on the abundance of fungal and bacterial C, N, and P cycling genes and their ratios, while significant effects of tree diversity were found for fungal C, fungal P, and bacterial C genes and the bacterial C:P gene ratio. We found no significant interactive effects (*P* < 0.05; Table 1). Further, two-way ANOVA at the plot mycorrhizal type mixture levels indicated the contribution of tree species identity to the fungal and bacterial genomic potential. The fungal C, N, and P cycling functional potentials were significantly affected by AM and AE mycorrhizal type plots, while C:N, C:P, and N:P genomic potentials were significant for AE and EcM plots (*P* < 0.05; Table S1). In contrast, the bacterial C, N, and P gene functional potentials and their ratios were significantly related to tree diversity levels in AM, AE, and EcM plots, except C and C:N gene ratios in AM tree species stands (*P* < 0.05; Table S2).

**TABLE 1:** Two-way ANOVA table showing the effects of plot mycorrhizal type (Plot_myco: AM, EcM, and AE) and tree diversity level (Div_levels: one, two, and four) on fungal and bacterial C, N, and P cycling genomic potential and their ratios (C:N, C:P, and N:P)^*a*^

Genomic potential	Variable	Fungi		Bacteria	
		F value	*P*	F value	*P*
C	Div_levels	16.29	0.001***	13.77	0.001**
	Plot_myco	38.99	0.001***	31.21	0.001**
	Plot_myco*div	5.245	0.056	3.167	0.083
N	Div_levels	8.079	0.180	3.558	0.060
	Plot_myco	1.804	0.003***	6.610	0.001**
	Plot_myco*div	2.610	0.075	1.708	0.182
P	Div_levels	7.254	0.008**	8.321	0.078
	Plot_myco	7.673	0.005**	70.77	0.001**
	Plot_myco*div	2.513	0.082	4.644	0.103
C: N	Div_levels	5.950	0.172	8.522	0.141
	Plot_myco	166.30	0.001***	21.16	0.003**
	Plot_myco*div	4.330	0.140	2.587	0.076
C:P	Div_levels	9.946	0.053	8.871	0.003**
	Plot_myco	91.417	0.001**	10.96	0.001**
	Plot_myco*div	4.824	0.161	1.828	0.162
N:P	Div_levels	8.499	0.062	1.539	0.216
	Plot_myco	103.8	0.003**	14.41	0.001***
	Plot_myco*div	0.664	0.515	1.686	0.187

^*a*^ Significant *P* values are annotated with significance levels: *P  ≤ 0.05, **P ≤ 0.01, and ***P ≤ 0.001.

### Relationship between microbial diversity and C, N, and P cycling genomic potential

**Fig 1:**
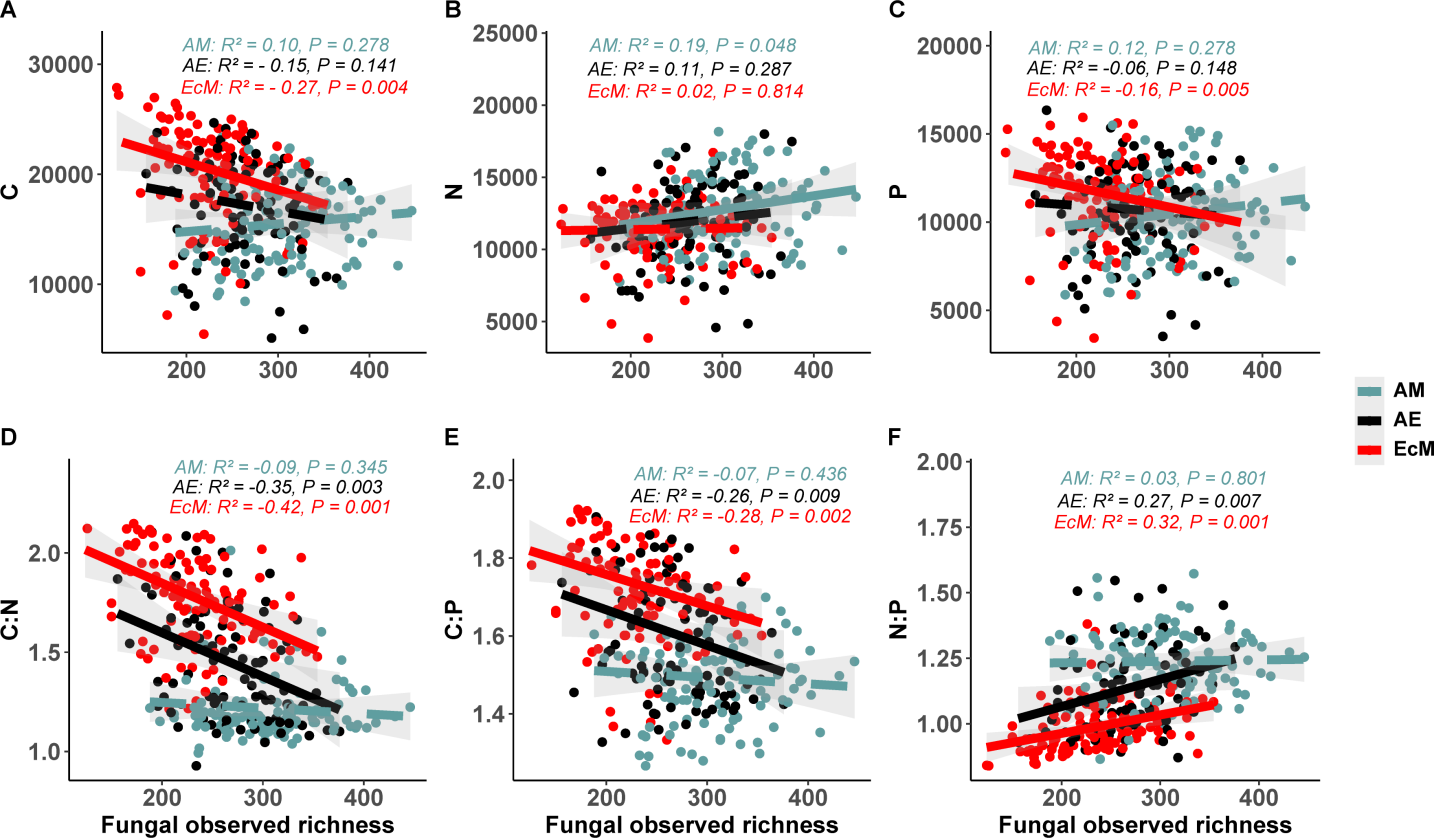
Relationships between fungal genomic functional potential (A) carbon; (B) nitrogen; (C) phosphorus; (D) carbon:nitrogen; (E) carbon:phosphorus; (F) nitrogen:phosphorus and fungal observed richness within AM, AE and EcM mycorrhizal type plots. The *r* and *P* values were calculated using a linear regression model. Regression lines are represented as solid for significant effects (*P* < 0.05) and dashed for non-significant effects (*P* > 0.05).

Linear regression analysis assessing the prediction of C, N, and P cycling genomic potentials from the fungal observed richness revealed a plot mycorrhizal type-specific relationship. A significant but negative relationship was observed for the C and P cycling genes with increasing fungal richness in EcM tree species plots, whereas a significant and positive relation was found between N cycling genomic potential and AM tree species plots (Fig. 1A through C). The C:N and C:P genomic potential ratios showed significant negative relationships with increasing fungal richness both in mono-EcM and mixed-AE plots, while the N:P genomic potential ratio showed a positive relationship (Fig. 1D through F). In contrast to the fungal community, no significant relationship was found between bacterial C, N, and P genomic potentials and their ratios and the bacterial observed richness in mono- and mixed-mycorrhizal type stands (Fig. S2).

### Relationship between microbial community and their C, N, and P cycling genomic potential

**Fig 2:**
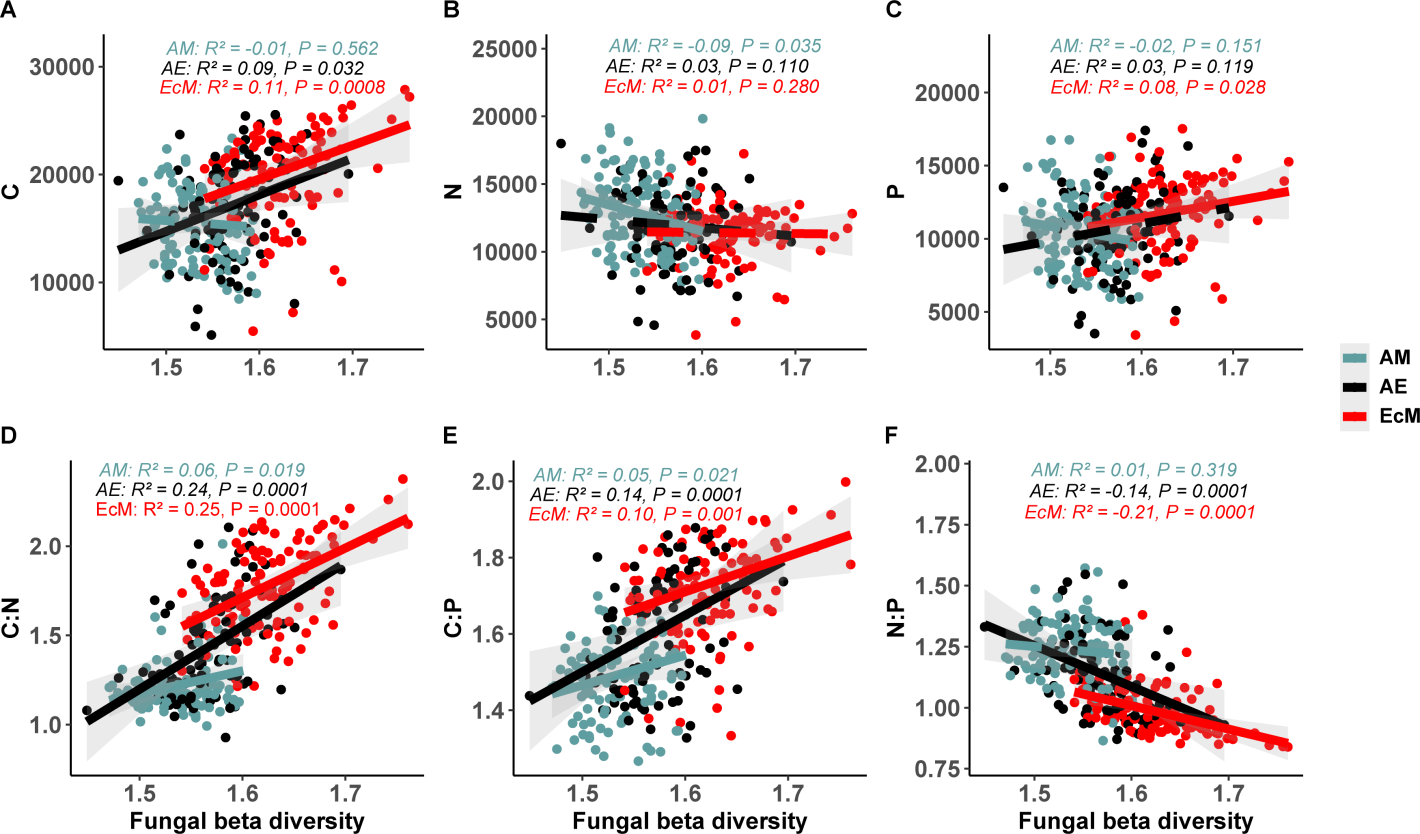
Relationships between fungal genomic functional potential (**A**) carbon; (**B**) nitrogen; (**C**) phosphorus; (**D**) carbon:nitrogen; (**E**) carbon:phosphorus; (**F**) nitrogen:phosphorus and fungal beta-diversity within AM, AE and EcM mycorrhizal type plots. The *r* and *P* values were calculated using a linear regression model. Regression lines are represented as solid for significant effects (*P* < 0.05) and dashed for non-significant effects (*P* > 0.05).

**Fig 3:**
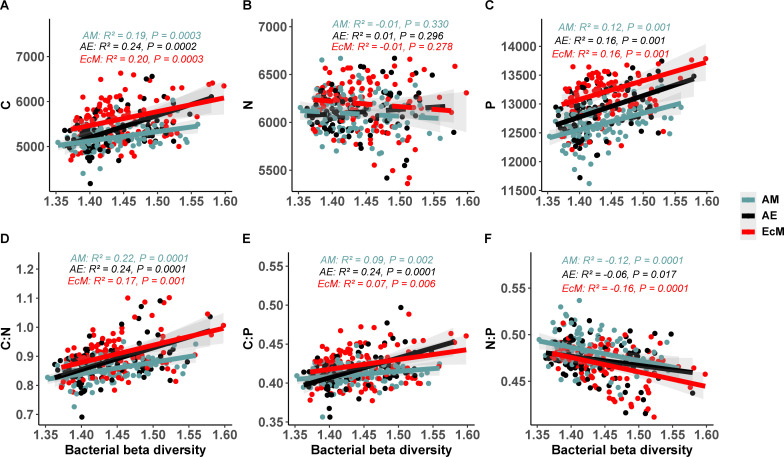
Relationships between bacterial genomic functional potential (A) carbon; (B) nitrogen; (C) phosphorus; (D) carbon:nitrogen; (E) carbon:phosphorus; (F) nitrogen:phosphorus and bacterial beta-diversity within AM, AE and EcM mycorrhizal type plots. The *r* and *P* values were calculated using a linear regression model. Regression lines are represented as solid for significant effects (*P* < 0.05) and dashed for non-significant effects (*P* > 0.05).

Similar regression analysis of C, N, and P cycling genomic potential predictions from the fungal beta-diversity also revealed a plot mycorrhizal type-specific relationship. A significant and positive relationship was observed for the C and P cycling genes with increasing fungal beta-diversity in EcM tree species plots. A similar result was found for C genomic potential in AE plots (Fig. 2A through C). Consistently, the C:N and C:P genomic potential ratios showed significant and positive relationships with increasing fungal beta-diversity both in mono-EcM and mixed-AE plots, while the N:P genomic potential ratio showed a negative relationship (Fig. 2D through F). Interestingly, both C and P cycling genomic potentials exhibited a significant and positive relationship with increasing bacterial beta-diversity in all the mono- and mixed-mycorrhizal type plots (Fig. 3A through C). The C:N, C:P, and N:P genomic potential ratios follow similar patterns as in fungi—positive for C:N and C:P and negative for N:P genomic potential ratios across AM, EcM, and mixed AE plots (Fig. 3D through F).

### Correlation between dominant microbial taxa and the microbial C, N, and P cycling genomic potential

**Fig 4:**
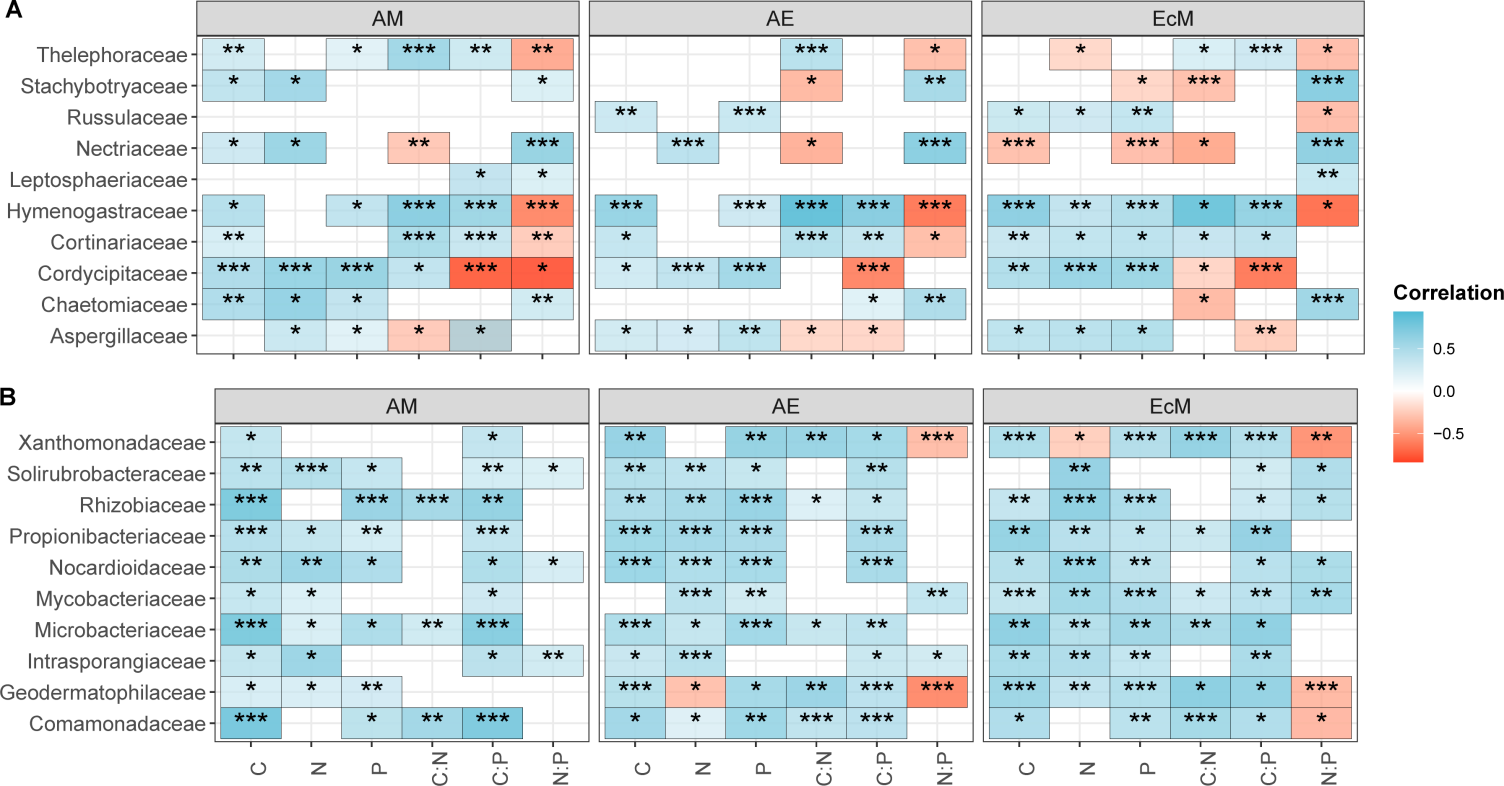
Correlations between the relative abundance of the dominant fungal (**A**) and bacterial (**B**) family and microbial genomic functional potential within mono- and mixed-mycorrhizal type. The red color indicates negative correlations, while the sky-blue color indicates positive correlations as presented on the correlation coefficients scale to the right.

Correlation analyses between the relative abundances of the ten most prevalent fungal and bacterial families and the genomic potentials for microbial C, N, and P cycling (as well as their ratios) revealed contributions related to both plot mycorrhizal type (Fig. 4) and tree diversity (Fig. S3). Among fungi, *Hymenogastraceae*, *Cordycipitaceae*, and *Aspergillaceae* were significantly associated with C, N, and P cycling genomic potentials across both mono- and mixed-mycorrhizal type plots, with notable decreases in *Aspergillaceae* contributions in two- and four-species mixture ECM plots. For bacteria, most dominant families showed significant correlations with microbial functional potential under all mycorrhizal plot types. Exceptions included *Xanthomonadaceae*, *Solirubrobacteraceae*, *Propionibacteriaceae*, *Mycobacteriaceae*, and *Geodermatophilaceae,* which did not show significant contributions in AM and ECM monocultures, AM two-species mixtures, or AE four-species stands. Overall, the dominant bacterial communities generally contributed significantly to C, N, and P cycling potentials at varying tree diversity levels within each plot mycorrhizal types (Fig. S3).

### Relationship between soil C:N:P stoichiometry and microbial C, N, and P cycling genomic potential

Regression analyses to test the relationship between soil C:N:P stoichiometry and the microbial C, N, and P cycling genomic potential at the plot mycorrhizal level showed a very weak and non-significant relationships for both fungi and bacteria (Table S3). However, similar analysis at tree diversity level within the respective plot mycorrhizal types indicated significant relationships between soil C and genomic C, soil C:P and genomic C:P in AM monocultures, and soil C:N and genomic C:N in EcM monocultures (Table S4, Fungi). In the case of the bacterial community, we only found a significant relationship between soil N and genomic N in four-species mixtures of the mixed-mycorrhizal type AE plot (Table S4, Bacteria). Furthermore, regression analysis testing the prediction of the microbial C, N, and P genomic potential from the soil C:N:P stoichiometry-based dissimilarity within plot mycorrhizal types revealed no significant relationships, except for bacterial N and P cycling genomic potential within the mixed and EcM mycorrhizal type stands, respectively (Fig. S4 and S5).

## DISCUSSION

In this study, we investigated the interplay of tree mycorrhizal type, mycorrhizal type mixture, tree diversity, and tree species identity in influencing the microbial genomic potential for carbon (C), nitrogen (N), and phosphorus (P) cycling in a controlled tree diversity experiment. Our findings indicated consistent and significant effects of plot mycorrhizal type and tree species identity on the fungal and bacterial genomic potential. We observed a clear relationship between fungal and bacterial diversity and community composition and their respective genomic potentials for C, N, and P cycling across both mono- and mixed-mycorrhizal type plots. Additionally, the relative contribution of dominant fungal and bacterial taxa to these cycling functions exhibited distinct patterns based on mycorrhizal type and tree diversity within plots. At the plot mycorrhizal level, no significant association was found between soil C:N:P stoichiometry or dissimilarity and the microbial genomic potentials, with the exception of bacterial N cycling potential, which showed a significant relationship in mixed-mycorrhizal type plots. However, when analyzed at the tree diversity level, significant relationships emerged between soil C:N:P ratios and microbial genomic potentials. Notably, fungal C cycling potential and the fungal C:P and C:N ratios were significantly related in AM monocultures, while bacterial N cycling potential was significantly associated in the mixed mycorrhizal (AE) four-species mixture stands.

### Microbial genomic potential is related to tree mycorrhizal type mixture and tree diversity levels

Our results indicate that microbial genomic functional potential varies in mono-AM, EcM, and mixed-AE mycorrhizal type plots. These variations are likely driven by the distinct nutrient acquisition strategies of AM- and EcM-associated fungi, which in turn shape the specialized functional adaptations of their co-existing microbial communities (19, 28). AM fungi, through their extraradical mycelium, extend the phosphorus depletion zone around the plant roots and primarily enhance plant phosphorus uptake by secreting organic acids and phosphatases that dissolve insoluble phosphorus compounds in the soil or by forming associations with phosphate-solubilizing microorganisms (28). This explains the significant differences in P cycling genomic potential observed in AM plots (Table S3). Similarly, EcM fungi extend the soil nutrient depletion zone and enhance primarily the uptake of nitrogen and phosphorus from soil and organic matter. ECM fungi possess complex extracellular enzymes (e.g., proteases and phosphatases) that break down complex organic compounds in soil organic matter releasing nutrients, such as nitrogen, phosphorus, and carbon. The co-existing bacteria also have the ability to fix atmospheric nitrogen, solubilize phosphate, and mineralize organic nutrients, thereby increasing nutrient availability in the rhizosphere that enables access to a broader nutrient spectrum, including organic forms of nitrogen and carbon (28, 33). Thus, the enhanced ability of EcM-associated rhizosphere microbiomes to decompose organic matter and mobilize carbon, nitrogen, and phosphorus likely accounts for the distinct patterns observed in fungal (C, N, and C:N) and bacterial (C, N, and P, C:N, C:P, and N:P) genomic potentials and their corresponding ratios within EcM plots (Table S2 and S3).

Beside the consistent effect of plot mycorrhizal type on the microbial C, N, and P cycling genomic potential, tree diversity also significantly influenced fungal (C and P cycle) and bacterial (C cycle and C:P ratio) genomic potentials but showed no effect within the plot mycorrhizal types. This could partly be explained by the fact that higher tree diversity has been reported to promote convergence of fungal and bacterial communities in rhizosphere soils (5, 6). As tree diversity increases, particularly in multi-species mixtures, the differences in resources provided by individual tree species or their mycorrhizal associations become less distinct (6). Thus, unlike monocultures, diverse tree species contribute a broader spectrum of carbon sources and nutrients through varied root exudates and leaf litter (34), supporting microbial taxa associated with multiple tree types and diminishing the dominance of specialist microbes linked to a single species or mycorrhizal association. This fosters complementary interactions and functional complementarity among microbial communities, leading to reduced impact of tree diversity on the microbial C, N, and P cycling genomic potential (31).

### Microbial diversity predicts microbial genomic functional potential

A growing number of studies demonstrated that soil C, N, and P concentrations and their combination can be considered as the main driver of soil microbial diversity (8, 35, 36). However, our study shifts this focus by examining the relationship between microbial diversity and microbial C, N, and P cycling genomic potential. Our findings highlight a positive link between fungal richness and fungal N cycle genes in AM plots, reflecting the greater saprotrophic diversity observed and reinforcing earlier reports that AM-dominated stands foster saprotrophic fungi that play central roles in litter decomposition and nitrogen mineralization (5, 28). Therefore, in AM soils, saprotrophic fungi play a central role in decomposition and nitrogen release, compensating for the limited intrinsic decomposition ability of AM fungi. This, in turn, contributes to the observed higher microbial nitrogen-cycling potential within these systems (37, 38). However, in EcM plots, greater fungal richness corresponded to lower abundances of C and P cycle genes, likely reflecting functional redundancy and niche complementarity among EcM fungi, where overlapping enzymatic traits reduce overall gene abundance despite higher taxonomic diversity (39).

The relationship between fungal diversity and genomic C:N, C:P, and N:P ratios showed that higher diversity in AE and EcM plots coincided with decreasing fungal C cycling genes, but relatively more N than P cycling genes. This suggests that in stands where EcM trees occur alone or co-exist with AM trees, higher fungal diversity is associated with functional shifts away from carbon cycling toward nitrogen cycling, with nitrogen processes outweighing phosphorus cycling (40, 41). These patterns reflect the fungal community adaptive response to the prevailing nutrient limitations in these ecosystems (42, 43). In contrast to fungal diversity, bacterial diversity showed no significant correlations with functional gene potential. This discrepancy likely stems from the high functional redundancy within bacterial communities, where many taxa share overlapping metabolic capabilities, resulting in a decoupling of taxonomic diversity and functional potential (40, 44). Furthermore, environmental filtering based on genome size and methodological limitations in inferring functional profiles may further mask any association between bacterial richness and functional genomic potential (45, 46).

### Microbial community composition predicts microbial genomic functional potential

In contrast to within-plot richness, greater fungal beta-diversity was linked to higher abundances of C cycling genes relative to N and P cycling genes, along with an increase in P over N cycling genes. These patterns suggest that more heterogeneous fungal communities across plots promote a broader and more active suite of carbon-degrading taxa, likely reflecting adaptations to break down diverse substrates such as cellulose and lignin using varied enzymatic systems (39). Concurrently, the greater prevalence of P cycling genes compared to N cycling genes in communities with higher fungal beta-diversity implies increased fungal communities with phosphorus solubilization and acquisition capacities, possibly driven by spatial variation in soil phosphorus availability or environmental conditions (41, 47).

Consistently increasing fungal beta-diversity was associated with higher genomic C:N and C:P potential ratios and a decline in the N:P ratio. This pattern reflects functional complementarity in carbon-processing enzyme, as distinct fungal communities across plots deploy varied extracellular enzyme toolkits (e.g. laccases for lignin degradation, cellulases for cellulose breakdown), thereby boosting the abundance of carbon-processing genes and increasing the C:N and C:P genomic ratios  (48). Conversely, nutrient acquisition strategies for N and P become spatially decoupled, reducing coordinated N:P gene expression across communities; for example, some plots may be dominated by chitinase-secreting taxa, while others are richer in phosphatase-producing fungi (42, 49). Moreover, variation in elemental stoichiometry among fungal guilds—specifically the higher C:N and C:P biomass ratios in saprotrophic fungi compared to mycorrhizal taxa— further explains the observed shifts in genomic potential ratios as fungal beta-diversity increases   (50). In contrast, higher fungal alpha diversity tends to be linked with a decrease in carbon cycling genes and a relative shift toward nitrogen-based functional potential. The bacterial beta-diversity also exhibited similar correlation patterns as the fungal beta-diversity, except for C, N, and P cycling genes and N:P genomic ratio in AM plots and P cycling genes in AE plots. Overall, elevated microbial beta-diversity fosters enhanced carbon-cycling capacity and a relative emphasis toward phosphorus cycling over nitrogen  (31, 51). These results underscore that community turnover, rather than richness *per se*, drives functional diversification in carbon, nitrogen, and phosphorus cycling among microbial communities  (42, 52).

Further analysis of the contribution of the relative abundances of the 10 most prevalent fungal and bacterial families to the microbial C, N, and P cycling genomic potentials and their ratios revealed contributions related to both plot mycorrhizal type (Fig. 4) and tree diversity levels (Fig. S3). The observed variation in the relative contributions of the fungal families (*Hymenogastraceae*, *Cordycipitaceae*, and *Aspergillaceae*) and bacterial families (*Xanthomonadaceae*, *Solirubrobacteraceae*, *Propionibacteriaceae*, *Mycobacteriaceae*, and *Geodermatophilaceae*) across tree diversity levels and plot mycorrhizal types could partially be associated with the functional guilds and metabolic potentials of members within these families.

### Relationships between soil C:N:P stoichiometry and soil microbial genomic potential

The significant correlations between soil C:N:P stoichiometry and microbial genomic potential, evident within specific mycorrhizal tree communities, underscore the role of plant community composition in shaping microbial nutrient cycling functions. In AM monocultures, the link between soil C and microbial genomic C reflects the role of AM fungi in promoting carbon inputs and microbial processing of carbon (20). Similarly, the correlation of soil C:N with microbial genomic C:N ratios in EcM monocultures reflects the well-established effect of ECM fungi on nitrogen acquisition and retention (28). In plots with mixed mycorrhizal associations, the responsiveness of bacterial communities to soil N level suggests that bacterial nutrient cycling is especially attuned to nitrogen availability in diverse plant communities (10). In contrast, the weak relationships at the broader plot mycorrhizal level likely result from spatial heterogeneity and complexity in soil and microbial communities, which may obscure more localized interactions (53). Collectively, these findings emphasize that microbial nutrient cycling potential is closely linked to soil stoichiometry under specific plant–mycorrhizal framework.

### Conclusion

Our findings underscore the pivotal role of mycorrhizal type, plot mycorrhizal mixture, tree species identity, and tree diversity in shaping the genomic potential of microbes involved in C, N, and P cycling. We also observed that these microbial functional potentials are intricately linked to microbial richness, community composition, and soil C:N:P stoichiometry. Importantly, our results indicate that shifts in microbial community composition, rather than richness alone, are key drivers of functional diversification in nutrient cycling. However, due to the limited availability of soil-based reference genome databases and the resulting constraints on functional assignments by PICRUSt2, future research should incorporate metagenomic approaches to provide more comprehensive insights.

Specifically, we demonstrate that interactions among diverse tree species, varied mycorrhizal types, and a rich microbial community collectively enhance microbial functionality in soils, leading to more efficient nutrient cycling. These insights are valuable for forest management, suggesting that planting mixtures of diverse tree species with different mycorrhizal associations, while promoting a robust soil microbial community, can improve forest resilience and productivity. Such management strategies could boost nutrient availability and cycling, ultimately supporting the development of healthier and more sustainable forest ecosystems.

## MATERIALS AND METHODS

### Experimental site and soil sampling

The MyDiv experimental platform is based at the Bad Lauchstädt Experimental Research Station of the Helmholtz Centre for Environmental Research-UFZ, Saxony-Anhalt, Germany (29). The climate features an average precipitation of 484 mm and an annual temperature of 8.8°C. The soil type is Haplic Chernozem, derived from Loess, with a pH of 7.4 (29). The experimental site was established in March 2015 on a former crop field, and the experiment comprises 80 plots measuring 11 × 11 m each, with a core area of 8 × 8 m in the center. Each plot contains 140 trees planted at 1-meter distance. The tree selection consists of 10 species, equally divided between AM and EcM groups and planted in one-, two-, and four-species mixtures. Additionally, treatments based on mycorrhizal types are incorporated, including individual AM, EcM, or mixed AM and EcM tree species (29). Eight tree species representing both AM and EcM associations were selected for analysis with equal representation in the experimental design (Fig. S1). A soil sample from the target tree rooting zone was prepared by pooling and thoroughly mixing four soil cores, each taken at 10 cm depth using 2 cm diameter soil corers (Fig. S1). Pooled soil samples were collected from 78 plots from 8 target tree species, with 2 individuals per plot, mycorrhizal type, and tree richness level (4 from monocultures, 12 from two-species mixtures, and 24 from four-species mixtures), resulting in a total of 320 samples (Fig. S1). The soil samples were sieved through a 2 mm mesh to eliminate root fragments, and 30 g samples were stored at −20°C for subsequent DNA extraction.

### Soil nutrient analysis

A 20 g subsoil sample was collected for the analysis of total carbon (C) and nitrogen (N). The sample was initially dried at 60°C and then finely ground into a powder using a ball mill (MM400; Retsch, Haan, Germany). Following this, the sample was subjected to an additional drying process for 24 h and subsequently transferred into tin capsules for analysis. Total nitrogen content was determined using the Kjeldahl method with an autoanalyzer (SEAL Analytical, Germany), while total organic carbon (TOC) was measured using a TOC analyzer (Liqui TOC II; Elementar Analyses System, Germany). Phosphate was determined using ion exchange membranes (IEM), following protocols by (54).

### DNA extraction, quantification, and amplicon library preparation

Soil DNA was extracted using the DNeasy PowerSoil Kit (Qiagen, Hilden, Germany) according to the manufacturer’s instructions as described in (5). The concentration of the extracted DNA was then quantified using a NanoDrop spectrophotometer (Thermo Fisher Scientific, Dreieich, Germany). Bacterial 16S rRNA gene amplicons targeting the V4 region were generated for library preparation through PCR amplification using the universal primers 515f and 806r (55) both containing Illumina adapter overhangs. A two-step semi-nested PCR amplification process was used to prepare the fungal amplicon libraries. The initial amplification was done using the primers ITS1F (56) and ITS4 (57) followed by a second round of PCR using the fITS7 (58) and ITS4 primers, which also included Illumina adapter sequences. Following amplification, AMPure XP magnetic beads (Beckman Coulter, Krefeld, Germany) were employed to purify the PCR products and eliminate residual primers and by-products. Illumina Nextera XT indexing was conducted in a second PCR amplification to introduce sample-specific barcodes, following another purification step with AMPure XP beads to ensure the indexed libraries’ quality. Quantification of the purified libraries was performed using the Qubit dsDNA High Sensitivity Assay (Thermo Fisher Scientific) and then pooled in equimolar ratio aiming to achieve a target of 4 nM concentration. High-quality sequencing data for downstream analysis was obtained using the Illumina MiSeq platform with paired-end 300 bp reads, employing the MiSeq Reagent Kit v3 (Illumina, Inc., San Diego, CA, USA). Sequencing was conducted at the Helmholtz Centre for Environmental Research-UFZ in Leipzig, Germany.

### Bioinformatics workflow

For processing the raw amplicon sequencing data, Quantitative Insights Into Microbial Ecology (QIIME 2 2020.2) (59) tool was employed for bioinformatics analysis. Initially, the raw sequences were filtered and trimmed using Cutadapt (60) to remove any low-quality bases, adapters, and primers from the reads. Following filtration, sequences were denoised and grouped into amplicon sequence variants (ASVs) using the DADA2 (61). For taxonomic assignment, the classify-sklearn naive Bayes classifier (62) was applied against the Silva-132-99-515-806-nb-classifier for bacterial sequence, while the fungal sequences were classified using the UNITE-ver8-99-classifier-04.02.2020 database. The fungal and bacterial ASV matrices were filtered and rarefied to ensure uniform sequencing depth (5,827 fungal and 11,264 bacterial reads per sample), and the resulting taxonomy and ASV abundance files were imported into R (version 4.4.3) (63) for further analysis using the phyloseq package (64). To predict the functional potential of the microbial communities, PICRUSt2 was employed, allowing for the inference of metagenome functional potential based on the identified ASVs. To determine the microbial taxa present in mono- (AM, EcM) and mixed-mycorrhizal (AM + EcM, abbreviated as AE) type and tree species diversity levels, stringent filtering was applied to both fungal and bacterial data sets. The tree species diversity levels included one-species (e.g., AM1, EcM1), two-species (e.g., AM2, AE2, EcM2), and four-species mixtures (e.g., AM4, AE4, EcM4). Initially, taxa with an abundance below 0.3% of the mean total sequencing reads were first removed. Then, within each combination of mono- and mixed-tree mycorrhizal types and tree diversity levels, taxa were retained only if they occurred in at least 66% (two-thirds) of the samples in their respective data sets. The refined data served as input for the PICRUSt2 (Phylogenetic Investigation of Communities by Reconstruction of Unobserved States) (30), a software to predict metagenomic functional profiles. In PICRUSt2, representative ASV sequences from both bacteria and fungi were aligned to reference genome databases for 16S and ITS regions using hidden Markov models (HMMER), with a minimum alignment threshold of 0.7 to ensure inclusion of all taxa classified at the genus level. The aligned sequences were then assigned to a reference phylogeny using a maximum likelihood phylogenetic approach implemented with the Gappa and EPA-ng tools. Number of genes per gene family for fungal and bacterial ASVs was predicted based on Enzyme Commission (EC) numbers using the MetaCyc database (65). The resulting EC number predictions were further filtered to focus on genes involved in carbon, nitrogen, and phosphorus cycling, according to previous literature (31).

### Statistical analysis

All statistical analyses and figure plotting were carried out with R (version 4.4.3) (63). Alpha diversity metrics, such as observed richness, were calculated using the microbiome package (66). The genomic functional potential for C, N, and P nutrient cycling and their ratios was calculated based on the predicted C, N, and P cycling gene contents of the respective microbial community. For instance, the C:N potential indicates the balance between carbon (e.g., energy and organic matter processing) and nitrogen (e.g., essential for proteins and enzymes) processes. A high C:N potential suggests that carbon-processing taxa dominate, while a low ratio indicates nitrogen-processing taxa are more active. High C:N ratios can indicate nitrogen limitation. Thus, in the following, C:N potential refers to the potential of taxa involved in the C cycle relative to those in the N cycle. The same applies to C:P and N:P cycling potential. A type II ANOVA was conducted with the "aov" function from the base package to test the effects of (i) tree species richness and plot mycorrhizal type mixture (mono- or mixed-mycorrhizal types), and (ii) tree species richness and tree species identity on microbial genomic potential. Linear regression analysis between the microbial C, N, and P cycling genomic potential and their ratios with soil C:N:P stoichiometry and the microbial alpha and beta-diversity was performed using hillR, ggplot2, and ggpubr package (67–69). Additionally, a correlation between microbial functional potential and the relative abundances of dominant fungal and bacterial families was computed using the "taxa.env.cor" function within the microbiomeseq package (70).

## Data Availability

The sequenced data sets are available in the Sequence Read Archive (SRA) at the National Center for Biotechnology Information (NCBI). Bacterial data can be accessed at PRJNA1147626, and fungal data are at PRJNA1092870.

## SUPPLEMENTAL MATERIAL

Supplemental material spectrum.00295-25-s0001.docx Tables S1 to S4; Fig. S1 to S5.

ASM does not own the copyrights to Supplemental Material that may be linked to, or accessed through, an article. The authors have granted ASM a non-exclusive, world-wide license to publish the Supplemental Material files. Please contact the corresponding author directly for reuse.
