# Supplementary material for: Impacts of mycorrhizal types, tree diversity, and species identity on the soil microbial genomic functional potential in temperate forests
Source: Microbiol Spectr. 2025 Nov 3;13(12):e00295-25. doi: 10.1128/spectrum.00295-25 (PMC12671117; doi:10.1128/spectrum.00295-25)
Supplement: Supplemental material — Tables S1 to S4; Fig. S1 to S5. [file spectrum.00295-25-s0001.docx]

**Table S1**: Two-way-ANOVA table showing the effects of tree diversity and tree species identity at the plot mycorrhizal type (AM, EcM and AE) on fungal C, N, P cycling genes genomic functional potential and their ratios (C:N, C:P and N:P)

|  |  | **AM** |  | **AE** |  | **EcM** |  |
| --- | --- | --- | --- | --- | --- | --- | --- |
| **Genomic potential** | **Variable** | **F value** | ***P*** | **F value** | ***P*** | **F value** | ***P*** |
| C | Div_levels | 1.383 | 0.242 | 0.356 | 0.552 | 0.401 | 0.779 |
|  | Tree sp | 10.61 | **<0.001***** | 3.108 | **0.005 **** | 0.517 | 0.671 |
|  | Tree sp*div | 1.881 | 0.137 | 1.509 | 0.176 | 2.217 | 0.090 |
| N | Div_levels | 1.504 | 0.222 | 1.764 | 0.187 | 2.126 | 0.053 |
|  | Tree sp | 23.37 | **<0.001***** | 5.939 | **<0.001***** | 4.127 | **0.008 **** |
|  | Tree sp*div | 2.621 | 0.054 | 1.835 | 0.091 | 0.606 | 0.612 |
| P | Div_levels | 1.763 | 0.187 | 0.028 | 0.868 | 4.855 | 0.059 |
|  | Tree sp | 13.93 | **<0.001***** | 4.374 | **0.0003***** | **2.084** | 0.106 |
|  | Tree sp*div | 2.392 | 0.072 | 1.459 | 0.194 | 1.600 | 0.193 |
| C: N | Div_levels | 0.230 | 0.632 | 3.323 | 0.072 | 2.490 | 0.056 |
|  | Tree sp | 1.105 | 0.351 | 3.223 | **0.004 **** | 5.940 | **0.001 ***** |
|  | Tree sp*div | 1.092 | 0.356 | 1.576 | 0.154 | 1.386 | 0.201 |
| C:P | Div_levels | 0.195 | 0.660 | 3.646 | 0.059 | 1.899 | 0.080 |
|  | Tree sp | 1.566 | 0.202 | 3.574 | **0.002 **** | 4.999 | **0.002 **** |
|  | Tree sp*div | 1.341 | 0.265 | 1.031 | 0.416 | 2.535 | 0.060 |
| N:P | Div_levels | 1.002 | 0.319 | 1.848 | 0.177 | 0.279 | 0.165 |
|  | Tree sp | 0.502 | 0.681 | 2.925 | **0.008 **** | 4.948 | **0.002 **** |
|  | Tree sp*div | 0.540 | 0.656 | 1.680 | 0.125 | 3.416 | 0.052 |

Significant P values are annotated with significance levels: * for P ≤ 0.05, ** for P ≤ 0.01 and *** for P ≤ 0.001

**Table S2**: Two-way-ANOVA table showing the effects of tree diversity and tree species identity at the plot mycorrhizal type mixture levels (AM, EcM and AE) on bacterial C, N, P cycling genes genomic functional potential and their ratios (C:N, C:P and N:P)

|  |  | **AM** |  | **AE** |  | **EcM** |  |
| --- | --- | --- | --- | --- | --- | --- | --- |
| **Genomic potential** | **Variable** | **F value** | ***P*** | **F value** | ***P*** | **F value** | ***P*** |
| C | Div_levels | 0.759 | 0.385 | 3.869 | 0.052 | 4.806 | 0.055 |
|  | Tree sp | 2.427 | 0.069 | 4.876 | **0.001 ***** | 14.713 | **0.001 ***** |
|  | Tree sp*div | 2.292 | 0.082 | 1.014 | 0.820 | 3.950 | 0.060 |
| N | Div_levels | 1.112 | 0.294 | 0.317 | 0.574 | 2.448 | 0.067 |
|  | Tree sp | 4.560 | **0.004 **** | 4.370 | **0.001 ***** | 4.668 | **0.033 *** |
|  | Tree sp*div | 0.278 | 0.841 | 1.818 | 0.052 | 1.145 | 0.334 |
| P | Div_levels | 0.485 | 0.487 | 1.481 | 0.0739 | 1.695 | 0.172 |
|  | Tree sp | 10.78 | **<0.001***** | 3.262 | **0.004 **** | 20.95 | **<0.001***** |
|  | Tree sp*div | 2.978 | 0.054 | 2.099 | 0.052 | 2.266 | 0.085 |
| C: N | Div_levels | 0.012 | 0.912 | 2.228 | 0.087 | 4.676 | 0.054 |
|  | Tree sp | 1.471 | 0.226 | 12.810 | **<0.001***** | 12.781 | **<0.001***** |
|  | Tree sp*div | 0.249 | 1.326 | 4.047 | 0.076 | 3.116 | 0.093 |
| C:P | Div_levels | 0.661 | 0.418 | 2.518 | 0.116 | 6.466 | 0.050 |
|  | Tree sp | 5.363 | **0.001 **** | 5.588 | **<0.001***** | 10.247 | **0.001 **** |
|  | Tree sp*div | 1.530 | 0.057 | 1.360 | 0.088 | 4.917 | 0.073 |
| N:P | Div_levels | 0.295 | 0.588 | 3.212 | 0.054 | 1.303 | 0.256 |
|  | Tree sp | 8.984 | **<0.001***** | 11.275 | **<0.001***** | 6.431 | **0.001 ***** |
|  | Tree sp*div | 1.165 | 0.327 | 1.698 | 0.121 | 0.838 | 0.476 |

Significant P values are annotated with significance levels: * for P ≤ 0.05, ** for P ≤ 0.01 and *** for P ≤ 0.001

**Table S3:** Comparative regression analysis of soil nutrient variable and microbial genomic functional potential in both mono (AM, EcM) and mixed (AE) mycorrhizal type. Soil indicate soil nutrient variable and Genomic indicate genomic functional potential processes.

|  | **AM** |  |  |  | **AE** |  |  | **EcM** |  |
| --- | --- | --- | --- | --- | --- | --- | --- | --- | --- |
| **Fungi** | **Coefficient** | **R** | **P** | **Coefficient** | **R** | **P** | **Coefficient** | **R** | **P** |
| Soil C vs Genomic C | -2810.381 | 0.023 | 0.109 | -2319.185 | 0.006 | 0.460 | 2257.169 | 0.014 | 0.208 |
| Soil N vs Genomic N | -16587.325 | 0.009 | 0.322 | -33714.463 | 0.026 | 0.115 | 1425.223 | 0.000 | 0.903 |
| Soil P vs Genomic P | -295.777 | 0.004 | 0.526 | 762.537 | 0.018 | 0.192 | 31.122 | 0.000 | 0.929 |
| Soil C:N vs Genomic C:N | -0.020 | 0.013 | 0.225 | 0.012 | 0.001 | 0.721 | 0.014 | 0.004 | 0.525 |
| Soil C:P vs Genomic C:P | 0.008 | 0.015 | 0.193 | 0.000 | 0.000 | 0.987 | -0.003 | 0.002 | 0.640 |
| Soil N:P vs Genomic N:P | -0.037 | 0.002 | 0.630 | 0.038 | 0.000 | 0.861 | -0.059 | 0.009 | 0.321 |
| **Bacteria** | **Coefficient** | **R** | **P** | **Coefficient** | **R** | **P** | **Coefficient** | **R** | **P** |
| Soil C vs Genomic C | -22.620 | 0.000 | 0.844 | 488.367 | 0.024 | 0.131 | 204.161 | 0.011 | 0.282 |
| Soil N vs Genomic N | 2253.356 | 0.032 | 0.059 | 2487.817 | 0.029 | 0.095 | 1032.066 | 0.004 | 0.502 |
| Soil P vs Genomic P | -33.556 | 0.004 | 0.517 | 25.356 | 0.001 | 0.774 | 38.608 | 0.004 | 0.531 |
| Soil C:N vs Genomic C:N | -0.006 | 0.021 | 0.127 | 0.004 | 0.002 | 0.672 | 0.012 | 0.029 | 0.071 |
| Soil C:P vs Genomic C:P | -0.001 | 0.005 | 0.449 | -0.004 | 0.022 | 0.149 | 0.000 | 0.001 | 0.790 |
| Soil N:P vs Genomic N:P | -0.008 | 0.004 | 0.510 | -0.016 | 0.004 | 0.524 | 0.017 | 0.018 | 0.163 |

Significant P values are annotated with significance levels: * for P ≤ 0.05, ** for P ≤ 0.01 and *** for P ≤ 0.001

**Table S4**: Comparative regression analysis of soil nutrient variable and microbial genomic functional potential in tree species richness (1sp, 2sp 4sp) in both mono (AM, EcM) and mixed (AE) mycorrhizal type. Soil indicate soil nutrient variable and Genomic indicate genomic functional potential processes.

| **Fungi** |  |  |  |  |  |  |
| --- | --- | --- | --- | --- | --- | --- |
| **AM** | **1sp** |  | **2sp** |  | **4sp** |  |
|  | **R^2^** | **P** | **R^2^** | **P** | **R^2^** | **P** |
| Soil C vs Genomic C | 0.290 | **0.031** | 0.009 | 0.590 | 0.015 | 0.329 |
| Soil N vs Genomic N | 0.002 | 0.856 | 0.017 | 0.472 | 0.007 | 0.487 |
| Soil P vs Genomic P | 0.006 | 0.762 | 3.030 | 0.976 | 0.0003 | 0.886 |
| Soil C:N vs Genomic C:N | 0.010 | 0.699 | 0.021 | 0.421 | 0.008 | 0.470 |
| Soil C:P vs Genomic C:P | 0.322 | **0.021** | 0.008 | 0.610 | 0.009 | 0.445 |
| Soil N:P vs Genomic N:P | 0.214 | 0.070 | 0.0009 | 0.864 | 0.016 | 0.317 |
| **AE** | **R^2^** | **P** | **R^2^** | **P** | **R^2^** | **P** |
| Soil C vs Genomic C |  |  | 0.035 | 0.304 | 0.003 | 0.883 |
| Soil N vs Genomic N |  |  | 0.016 | 0.489 | 0.031 | 0.160 |
| Soil P vs Genomic P |  |  | 0.045 | 0.240 | 0.005 | 0.558 |
| Soil C:N vs Genomic C:N |  |  | 0.0003 | 0.916 | 0.003 | 0.649 |
| Soil C:P vs Genomic C:P |  |  | 0.0005 | 0.898 | 0.003 | 0.664 |
| Soil N:P vs Genomic N:P |  |  | 0.0004 | 0.904 | 0.007 | 0.492 |
| **EcM** | **R^2^** | **P** | **R^2^** | **P** | **R^2^** | **P** |
| Soil C vs Genomic C | 0.022 | 0.580 | 0.048 | 0.225 | 0.002 | 0.709 |
| Soil N vs Genomic N | 0.009 | 0.725 | 2.04E.0 | 0.980 | 0.006 | 0.542 |
| Soil P vs Genomic P | 0.059 | 0.362 | 0.008 | 0.617 | 2.41E.0 | 0.990 |
| Soil C:N vs Genomic C:N | 0.381 | **0.010** | 0.0834 | 0.108 | 0.001 | 0.795 |
| Soil C:P vs Genomic C:P | 0.001 | 0.876 | 0.062 | 0.165 | 0.005 | 0.545 |
| Soil N:P vs Genomic N:P | 0.009 | 0.715 | 0.015 | 0.494 | 0.018 | 0.279 |
| **Bacteria** |  |  |  |  |  |  |
| **AM** | **1sp** |  | **2sp** |  | **4sp** |  |
|  | **R^2^** | **P** | **R^2^** | **P** | **R^2^** | **P** |
| Soil C vs Genomic C | 0.006 | 0.758 | 0.002 | 0.806 | 0.006 | 0.531 |
| Soil N vs Genomic N | 0.0006 | 0.925 | 0.048 | 0.227 | 0.034 | 0.141 |
| Soil P vs Genomic P | 0.076 | 0.298 | 0.057 | 0.185 | 0.035 | 0.136 |
| Soil C:N vs Genomic C:N | 0.152 | 0.134 | 0.001 | 0.816 | 0.032 | 0.154 |
| Soil C:P vs Genomic C:P | 0.044 | 0.431 | 0.121 | 0.050 | 0.021 | 0.243 |
| Soil N:P vs Genomic N:P | 0.151 | 0.136 | 0.028 | 0.357 | 0.018 | 0.285 |
| **AE** | **R^2^** | **P** | **R^2^** | **P** | **R^2^** | **P** |
| Soil C vs Genomic C |  |  | 2.95E.0 | 0.976 | 0.047 | 0.084 |
| Soil N vs Genomic N |  |  | 0.0002 | 0.928 | 0.090 | **0.016** |
| Soil P vs Genomic P |  |  | 0.018 | 0.453 | 0.003 | 0.626 |
| Soil C:N vs Genomic C:N |  |  | 0.010 | 0.568 | 0.013 | 0.358 |
| Soil C:P vs Genomic C:P |  |  | 0.055 | 0.314 | 0.0002 | 0.899 |
| Soil N:P vs Genomic N:P |  |  | 0.085 | 0.105 | 0.017 | 0.293 |
| **EcM** | **R^2^** | **P** | **R^2^** | **P** | **R^2^** | **P** |
| Soil C vs Genomic C | 0.0002 | 0.952 | 0.100 | 0.076 | 0.004 | 0.599 |
| Soil N vs Genomic N | 0.032 | 0.506 | 0.076 | 0.125 | 0.009 | 0.436 |
| Soil P vs Genomic P | 8.44E.0 | 0.973 | 0.089 | 0.096 | 0.037 | 0.123 |
| Soil C:N vs Genomic C:N | 0.107 | 0.215 | 0.037 | 0.285 | 0.011 | 0.393 |
| Soil C:P vs Genomic C:P | 0.185 | 0.102 | 0.111 | 0.062 | 1.14E.0 | 0.993 |
| Soil N:P vs Genomic N:P | 0.004 | 0.811 | 0.113 | 0.059 | 0.010 | 0.414 |

Significant P values are annotated with significance levels: * for P ≤ 0.05, ** for P ≤ 0.01 and *** for P ≤ 0.001


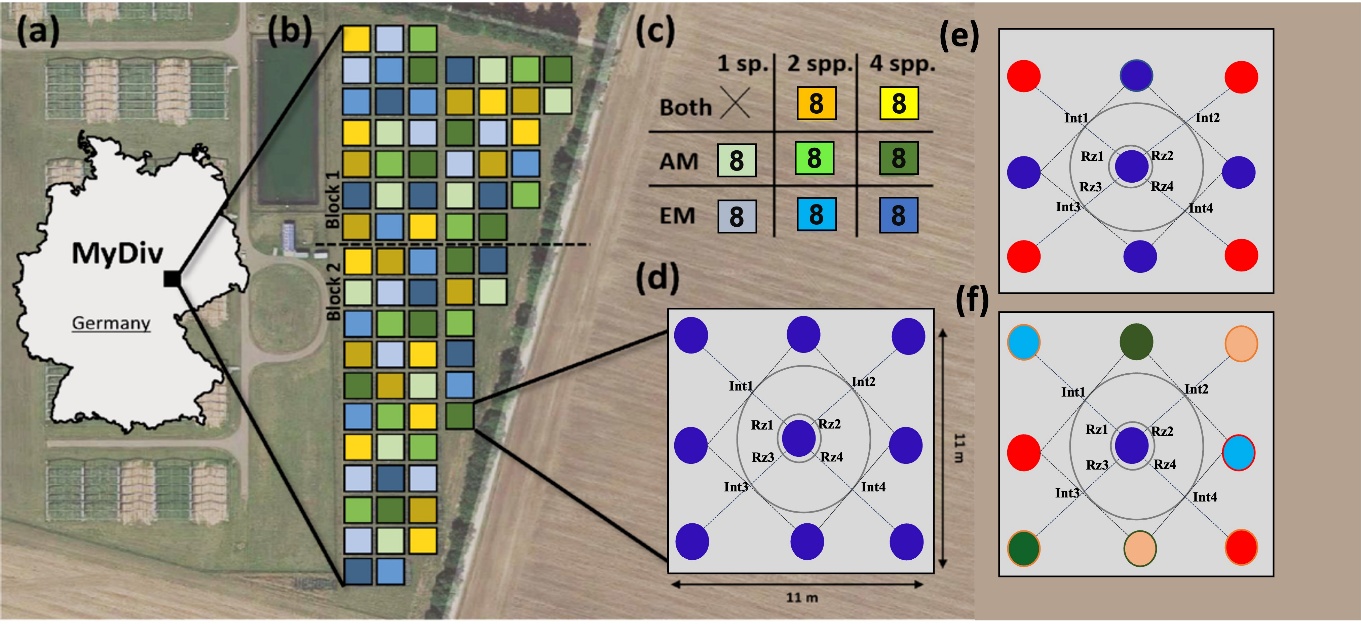


| **Mycorrhizal type** | **Tree species** | **Family** | **Mono** | **Two** | **Four** | **Total** |
| --- | --- | --- | --- | --- | --- | --- |
| AM | *Acer pseudoplatanus L.* | Sapindaceae | 4 | 12 | 24 | 40 |
|  | *Fraxinus excelsior L.* | Oleaceae | 4 | 12 | 24 | 40 |
|  | *Prunus avium L.* | Rosaceae | 4 | 12 | 24 | 40 |
|  | *Sorbus aucuparia L.* | Rosaceae | 4 | 12 | 24 | 40 |
| EcM | *Betula pendula Roth* | Betulaceae | 4 | 12 | 24 | 40 |
|  | *Carpinus betulus L.* | Betulaceae | 4 | 12 | 24 | 40 |
|  | *Quercus petraea Liebl.* | Fagaceae | 4 | 12 | 24 | 40 |
|  | *Fagus sylvatica L.* | Fagaceae | 4 | 12 | 24 | 40 |
|  |  |  | 32 | 96 | 192 | **320** |

**Figure S1:** The MyDiv experimental platform location (a). The plot layout with treatment varying in tree species richness (1, 2, 4 species) includes varying mycorrhizal types and their mixtures (AM, AE, EcM) (b). tree species per plots per richness and mycorrhizal combination (c). Sampling design within plots, pooled soil samples were collected from the rooting zone of the target tree (Rz1–Rz4) and from interaction zones between the target and surrounding trees (Int1–Int4). single-species (d), two-species (e), and four-species mixtures (f). The table shows the number of pooled samples per target tree species, plot mycorrhizal type, and diversity level. In monocultures, each species occurs in 2 plots, with 2 individuals sampled per plot (2 × 2 = 4 samples). In two-species mixtures, each species occurs in 6 plots (4 same mycorrhizal type, 2 AMF+EMF), with 2 individuals per plot (2 × 6 = 12 samples). In four-species mixtures, each species occurs in 12 plots (8 same mycorrhizal type, 4 AMF+EMF), again with 2 individuals per plot (2 × 12 = 24 samples). This totals 40 samples per species (4 + 12 + 24), yielding 320 pooled samples overall (8 species × 40) from target tree rooting and interactive rooting zones. Modified after (Ferlian et al., 2018; Haq et al., 2024; Haq et al., 2025)

**
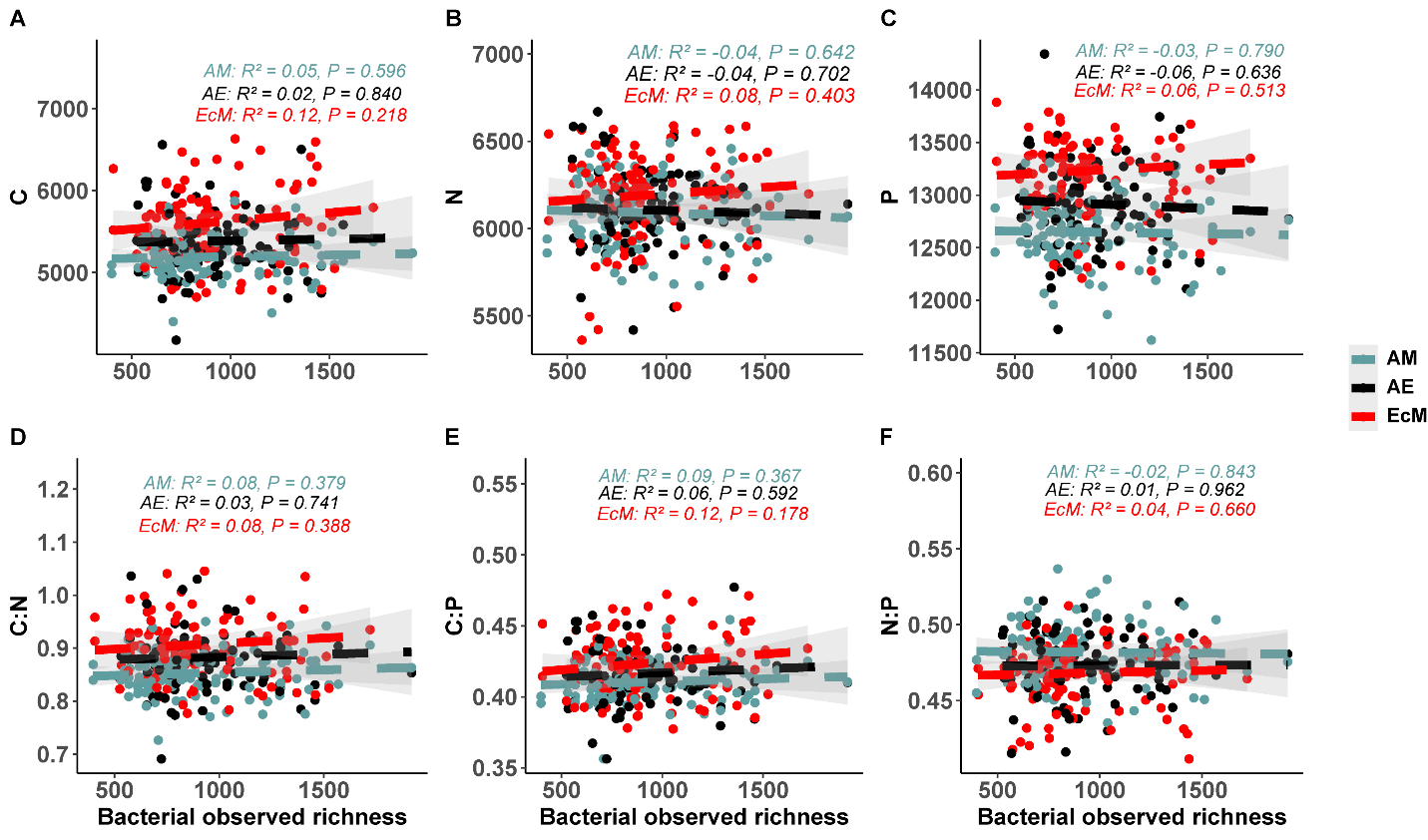
**

**Figure S2.** Relationships between bacterial genomic functional potential with bacterial observed richness within mono (AM, EcM) and mixed (AE) mycorrhizal type plots. The *r* and *p* values were calculated using a linear regression model. Regression lines are represented as solid for significant effects (p < 0.05) and dashed for non-significant effects (p > 0.05)

**
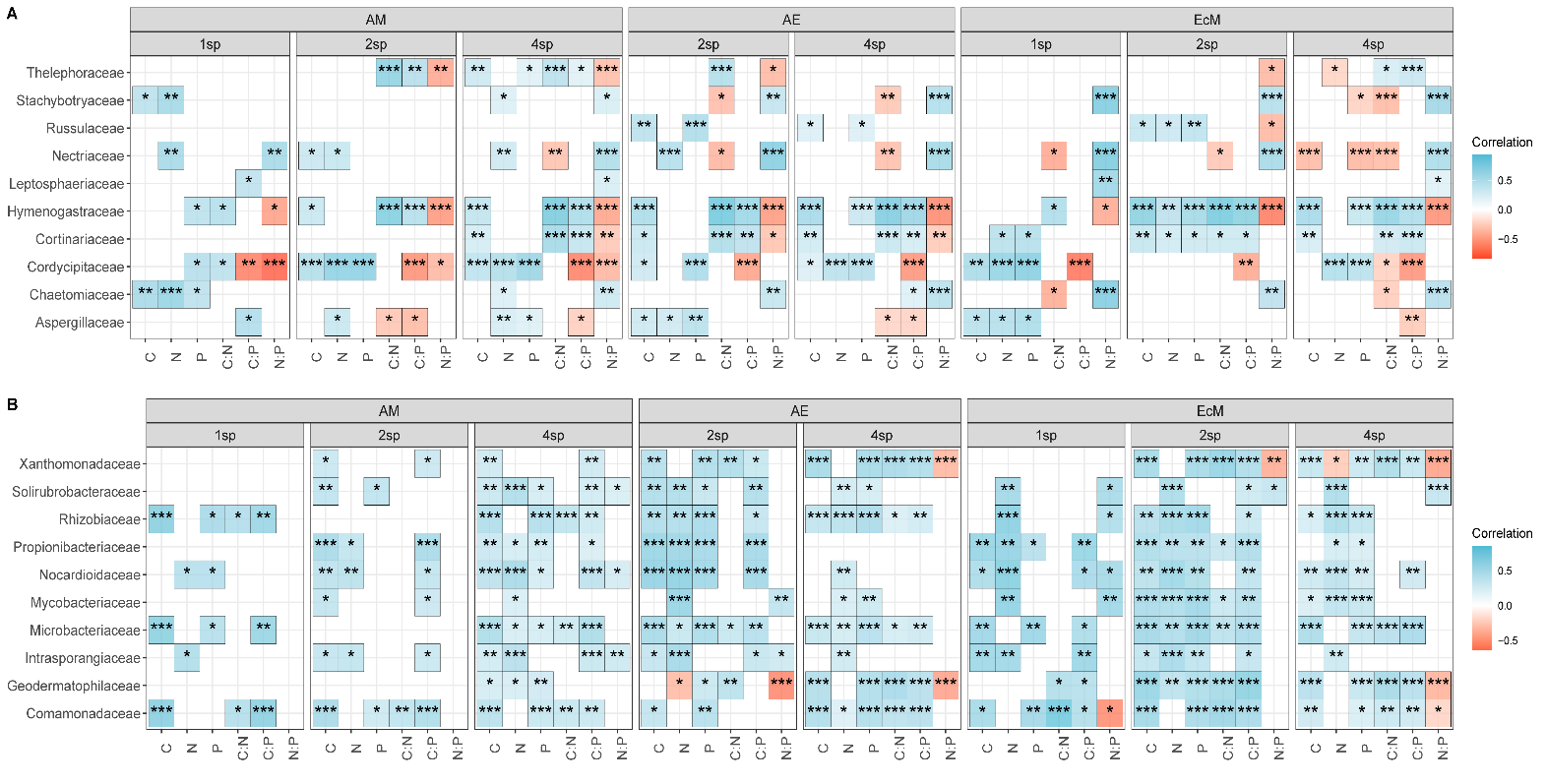
**

**Figure S3**. Spearman correlations between the relative abundance of the dominant fungal (A) and bacterial (B) families and microbial genomic functional potential in tree species richness (1sp, 2sp, 4sp) in both mono (AM, EcM) and mixed (AE) mycorrhizal type plots. The red color indicates negative correlations, while the sky-blue color indicates positive correlations.

**
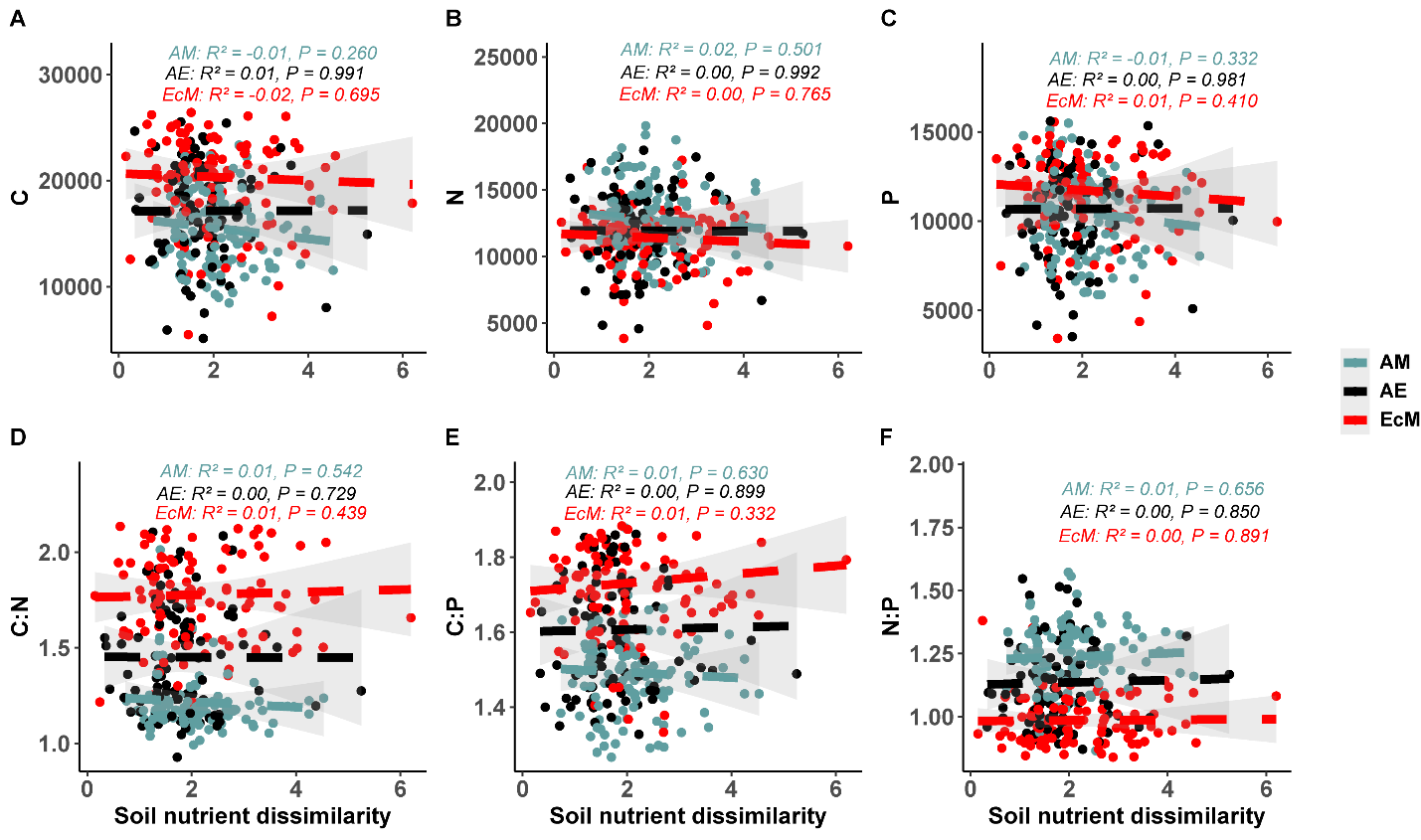
**

**Figure S4.** Relationships between fungal genomic functional potential with soil nutrient dissimilarity within mono (AM, EcM) and mixed (AE) mycorrhizal type plots. The *r* and *p* values were calculated using a linear regression model. Regression lines are represented as solid for significant effects (p < 0.05) and dashed for non-significant effects (p > 0.05)

**
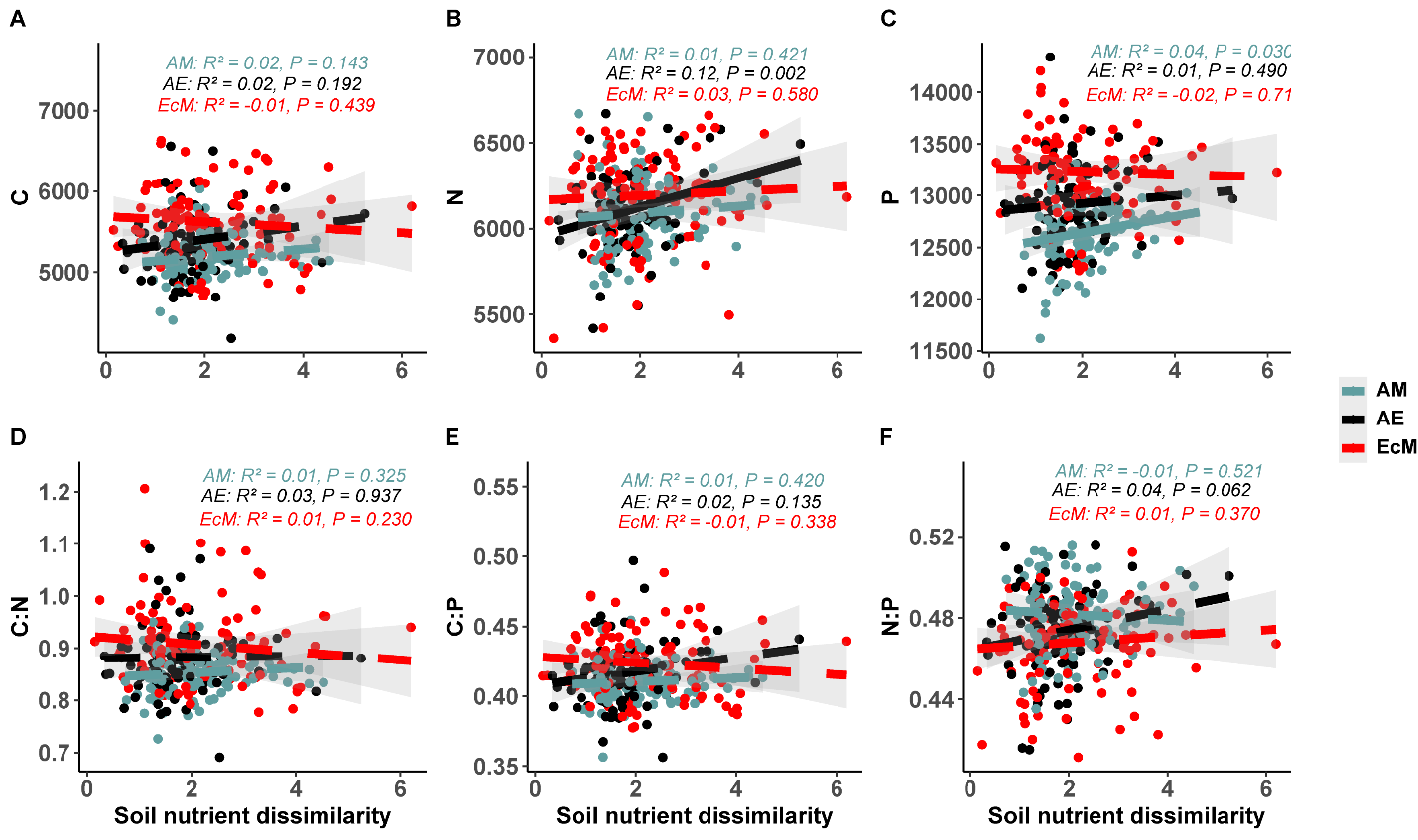
**

**Figure S5.** Relationships between bacterial genomic functional potential with soil nutrient dissimilarity within mono (AM, EcM) and mixed (AE) mycorrhizal type plots. The *r* and *p* values were calculated using a linear regression model. Regression lines are represented as solid for significant effects (p < 0.05) and dashed for non-significant effects (p > 0.05)
